# Implementation of a free smoking-cessation program in a Lebanese academic medical center

**DOI:** 10.18332/tid/125916

**Published:** 2020-09-08

**Authors:** Maya Romani, Rima Nakkash, Sarah Jawhar, Ramzi G. Salloum

**Affiliations:** 1 Department of Family Medicine, American University of Beirut Medical Center, Beirut, Lebanon; 2 Department of Health Promotion and Community Health, Faculty of Health Sciences, American University of Beirut, Beirut, Lebanon; 3 Department of Health Outcomes and Biomedical Informatics, College of Medicine, University of Florida, Gainesville, United States

**Keywords:** free clinic, smoking cessation, counseling

## Abstract

**INTRODUCTION:**

Despite the exceptionally high prevalence of tobacco use in Lebanon, few cessation programs exist. The American University of Beirut (AUB) developed one of the first smoking-cessation programs in the country in 2015, and the program became free-of-charge to patients in 2018. The program offers initial visit(s) with a primary care provider, in-person and/or telephone counseling, acupuncture, and medications.

**METHODS:**

We assessed patient characteristics, treatments used, and patient outcomes in the first year of implementing the free smoking-cessation program, compared to the original program. We compared 87 smokers who initiated treatment in the free program with 47 patients in the original program.

**RESULTS:**

At baseline, smokers in the free program were younger, smoked fewer cigarettes per day, and had lower CO levels than smokers in the original program. At 1 month follow-up, 72.9% were abstinent in the free program, compared with 42.2% in the original program (p<0.001). Smokers who had ≥2 primary care visits and those who had ≥1 acupuncture visits had higher rates of abstinence at 1 month and those who were prescribed bupropion had higher rates of abstinence at 12 months.

**CONCLUSIONS:**

Implementation of the free smoking cessation program demonstrates preliminary efficacy, with telephone support offering potential for scalability.

## INTRODUCTION

Lebanon has high smoking prevalence rates of combustible tobacco use compared to other middle-income countries, reaching 34% in males and 21% in females for daily cigarette smoking, and 26% and 24%, respectively, for waterpipe smoking^1^. The incidence of lung cancer in Lebanon is highest among females and second highest among males in the Eastern Mediterranean Region^2^. Despite the high burden of smoking in the Lebanese population, few resources exist to support smokers with tobacco cessation treatment^3^.

The American University of Beirut (AUB), a leading institution of higher education in Lebanon and the region, with an affiliated tertiary medical center, developed one of the first smoking-cessation programs in the country in 2015 in response to an identified need within the university community^4, 5^. The program offers counseling to promote learning behavior modification and provides nicotine replacement therapy (NRT), including nicotine gum and patch. Effective from 1 January 2018, AUB implemented a tobacco-free university policy. As part of the policy, the smoking cessation program became free-of-charge with the goal of increasing access to tobacco treatment services.

**Figure 1:**
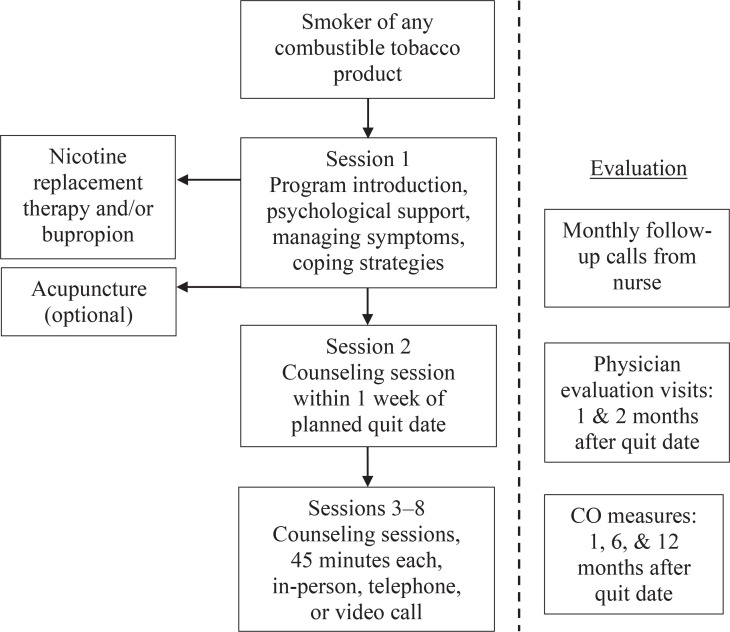
Smoking cessation program treatment flowchart

The free program accepts smokers of all combustible tobacco products, including cigarettes and waterpipe. The program is staffed with two physicians who are tobacco treatment specialists, two nurses specialized in behavioral smoking cessation counseling, and one administrator. Patients who enroll in the program receive up to eight sessions of counseling, as well as nicotine replacement therapy (NRT) free-of-charge (Figure 1). Patients with contraindication for NRT or with a co-occurring mental health condition (e.g. dysthymia, major depressive disorder) are prescribed bupropion instead of NRT (varenicline is not available in Lebanon). In addition, patients are offered the option of acupuncture due to its therapeutic role in suppressing addiction and reducing withdrawal symptoms^6^.

At the initial visit, the patient is introduced to the program, offered psychological support, provided information on managing withdrawal symptoms and coping strategies, and scheduled for a behavioral counseling session within one week of the planned quit date. The six remaining sessions lasting 45 minutes each are held on a weekly basis for two months with the nurse/smoking cessation specialist. Patients are requested to participate in these six sessions in person. If the patient is unable to visit the clinic, the session is held either via telephone or via a video call through the mobile application WhatsApp. Additionally, participants are encouraged to contact the nurse in case they experience any adverse drug reaction or need behavioral support and guidance through their cessation treatment process. In parallel with the weekly sessions, patients have two follow-up visits with the physician to evaluate withdrawal symptoms and potential medication side effects (at one and two months post quit-date). After completing eight weeks of treatment, patients are contacted by telephone on a monthly basis to ensure compliance and abstinence, and CO measures are collected at 1, 6, and 12 months. All program metrics are recorded in the patient’s electronic health record.

## METHODS

We conducted a retrospective study of patients treated in the first year of the free program, spanning from November 2018 to December 2019, compared with the original program (2015–2017). Patient characteristics at baseline included age, gender, tobacco product used at the initial visit (e.g. cigarettes, waterpipe), number of cigarettes per day, CO level, and the Fagerström Test for Nicotine Dependence (FTND) score^7^. Treatments received included behavioral counseling, acupuncture, and prescribed medications (i.e. bupropion, NRT, and combination bupropion + NRT). Outcomes included CO-verified abstinence at 1, 6, and 12 months after the set quit date. Descriptive statistics are reported, including mean and standard deviation for continuous variables, and frequency and percentage for categorical variables. Quit outcomes were compared between the original and free programs across all three follow-up periods using average treatment effects estimation with inverse-probability weights that adjusted for age and nicotine dependence. Comparison of patient baseline characteristics between the free and original programs were conducted using t-tests for continuous variables and chi-squared tests for categorical variables. Associations between treatment types and quit outcomes at 1, 6, and 12 months among participants in the free program were examined using chi-squared tests. Associations with p<0.05 were considered statistically significant. Data analysis was conducted in 2020 using Stata version 15.1 (StataCorp LLC). Ethical approval was acquired from the AUB Institutional Review Board.

## RESULTS

The analysis included 87 patients who completed the initial visit in the free program compared with 47 patients in the original program (Table 1). Among those who initiated treatment in the free program, mean age was 41.6 years (SD=15.0), 27 (31.0%) were female, 21 (41.1%) were light smokers (≤10 cigarettes/day), and 6 (6.8%) were waterpipe smokers. The mean CO level was 13.4 ppm (SD=6.7) and 34 (42.0%) scored a ≥7 on the FTND. Significant differences in patient characteristics were observed compared to the original program, including in mean age (50.0 years), cigarettes per day (none smoked ≤10 cigarettes), and CO level (20.5 ppm).

**Table 1:** Characteristics of patients participating in the free smoking-cessation program (2018–2019) and the original program (2014–2017)

*Characteristics*	*Free program (N=87) n (%)*	*Original program (N=47) n (%)*	*p*
**Age** (years), mean±SD	41.6±15.0	50.0±14.8	0.002
**Gender**			0.182
Male	60 (69.0)	27 (57.4)	
Female	27 (31.0)	20 (42.6)	
**Tobacco product(s) used**			0.263
Cigarettes	80 (92.0)	47 (100.0)	
Waterpipe	5 (5.7)	-	
Pipe	1 (1.1)	-	
Cigarettes + waterpipe	1 (1.1)	-	
**Cigarettes per day**			<0.001
≤10	21 (41.1)	-	
11–20	34 (39.0)	12 (28.6)	
21–30	11 (12.6)	18 (42.9)	
≥31	15 (17.2)	12 (26.6)	
Not a cigarette smoker	6 (6.9)	-	
**CO level**, mean±SD	13.4±6.7	20.5±2.1	<0.001
**Fagerström test for nicotine dependence**			0.221
<7	47 (58.0)	19 (46.3)	
≥7	34 (42.0)	22 (53.7)	

Overall, 62 (72.9%) were abstinent at 1 month in the free program, compared with 19 (42.2%) in the original program (p=0.006). No significant differences were observed at 6 and 12 months (Table 2). Among smokers participating in the free program, the 1-month abstinence rate was higher for those who had 2 or more primary care visits (93.8%) compared to those with only 1 visit (60.4%) and among those who had acupuncture (79.7%) compared to those who did not (57.7%). Additionally, abstinence at 12 months was higher among those who were prescribed bupropion only (83.3%) compared to NRT (14.3%) or combination therapy (33.3%).

**Table 2:** Smoking cessation treatment types and outcomes at 1, 6, and 12 months follow-up

*Treatments received*	*Abstinence 1 month*		*Abstinence 6 months*		*Abstinence 12 months*	
	*n (%)*	p*	*n (%)*	p*	*n (%)*	p*
**Smoking-cessation program**		0.006		0.532*		0.940*
Original program	19 (42.2)		15 (32.6)		11 (24.4)	
Free program	62 (72.9)		29 (44.6)		13 (34.2)	
*Free smoking-cessation program*	*Abstinence 1 month*		*Abstinence 6 months*		*Abstinence 12 months*	
	*n (%)*	*p*	*n (%)*	*p*	*n (%)*	*p*
**Number of physician visits**		0.001		0.077		0.305
1	32 (60.4)		16 (57.1)		8 (42.1)	
≥2	30 (93.8)		13 (35.1)		5 (26.3)	
**Number of counseling sessions**		0.889		0.204		0.728
<4	10 (71.4)		3 (27.3)		11 (35.5)	
≥4	52 (73.2)		26 (48.2)		2 (28.6)	
**Acupuncture sessions**		0.036		0.174		0.297
0	15 (57.7)		6 (31.6)		3 (23.1)	
≥1	47 (79.7)		23 (50.0)		10 (40.0)	
**Prescribed medications**		0.922		0.086		0.012
Bupropion	5 (71.4)		5 (83.3)		5 (83.3)	
Nicotine replacement therapy	30 (75.0)		10 (34.5)		2 (14.3)	
Combination therapy	27 (71.0)		14 (46.7)		6 (33.3)	

* Average treatment effects estimation using inverse-probability weights adjusting for age and nicotine dependence.

## DISCUSSION

In its initial year, the free program demonstrated a high level of effectiveness and has expanded the reach of the original program, including among younger and light smokers. Smokers in the free cessation program had significantly higher abstinence rates at 1 month follow-up compared with their counterparts who had participated in the original program. This finding is consistent with prior evidence of the beneficial effects of removing financial barriers for smoking cessation treatment^8^.

Among smokers who participated in the free smoking-cessation program, we found higher abstinence rates in those who had more than one physician visit, those who participated in acupuncture, and those treated with bupropion. Delivery of physician advice to smokers is a critical component of clinical practice guidelines for smoking cessation^9^, and therefore it is not surprising that smokers who had more than one physician encounter were more likely to quit successfully. In addition, smokers who participated in acupuncture had higher abstinence rates. This result is consistent with a recent systematic review that evaluated the evidence in support of the beneficial effect of acupuncture on smoking cessation^6^. Also, our findings suggest that bupropion may be more effective than NRT in supporting patients to maintain long-term abstinence. Although our study did not document mental health diagnoses, patients were more likely to be prescribed bupropion over NRT if they had co-occurring mental health conditions. Given emerging evidence that bupropion may be more beneficial than NRT for smokers with a history of depression^10^, and the increasing public health concern about mental health in Lebanon^11, 12^, this finding has important practice implications on prescribing cessation medications as a part of tobacco use treatment in Lebanon and beyond.

Although the program has enjoyed exceptional success in the Lebanese context, where few resources exist to support smokers with cessation services, it has thus far operated at a small scale. Efforts are needed to scale up the program to meet the unmet needs of smokers, who represent a large portion of the Lebanese population. Telephone-based counseling services (i.e. quit lines) should be considered in this regard, as they are a recommended solution for delivering population-based smoking cessation support, especially in low-resource settings^13^.

In addition, while the vast majority of patients in the program were cigarette smokers, the prevalence of waterpipe smoking in Lebanon is comparable to the prevalence of cigarette smoking^1^. There are many patient-level barriers to waterpipe smokers seeking cessation support, including misperceptions about harm, as well as the social and cultural norms associated with the practice. From a clinical point of view, a recent survey of Lebanese primary care practitioners found they were more likely to counsel patients against cigarette smoking than waterpipe smoking, underscoring a lack of knowledge about waterpipe smoking cessation techniques^14^.

## CONCLUSIONS

Implementation of the free smoking-cessation program demonstrates preliminary efficacy, with telephone support offering potential for scalability. Future enhancements to the program should also consider strategies to facilitate the delivery of smoking cessation services to waterpipe smokers.

## References

[1] Chaaya M, Nakkash R, Saab D, Kadi L, Afifi R (2019). Effect of tobacco control policies on intention to quit smoking cigarettes: A study from Beirut, Lebanon. Tob Induc Dis.

[2] Temraz S, Charafeddine M, Mukherji D, Shamseddine A (2017). Trends in lung cancer incidence in Lebanon by gender and histological type over the period 2005-2008. J Epidemiol Glob Health.

[3] Pine-Abata H, McNeill A, Murray R, Bitton A, Rigotti N, Raw M (2013). A survey of tobacco dependence treatment services in 121 countries. Addiction.

[4] Chaaya M, Alameddine M, Nakkash R, Afifi RA, Khalil J, Nahhas G (2013). Students' attitude and smoking behaviour following the implementation of a university smoke-free policy: a cross-sectional study. BMJ Open.

[5] Salloum RG, Abbyad CW, Kohler RE, Kratka AK, Oh L, Wood KA (2015). Assessing preferences for a university-based smoking cessation program in Lebanon: a discrete choice experiment. Nicotine Tob Res.

[6] Wang JH, van Haselen R, Wang M (2019). Acupuncture for smoking cessation: A systematic review and meta-analysis of 24 randomized controlled trials. Tob Induc Dis.

[7] Heatherton TF, Kozlowski LT, Frecker RC, Fagerstrom KO (1991). The Fagerstrom Test for Nicotine Dependence: a revision of the Fagerstrom Tolerance Questionnaire. Br J Addict.

[8] Reda AA, Kotz D, Evers SM, van Schayck CP (2012). Healthcare financing systems for increasing the use of tobacco dependence treatment. Cochrane Database Syst Rev.

[9] The Clinical Practice Guideline Treating Tobacco Use and Dependence 2008 Update Panel, Liaisons, and Staff (2008). A Clinical Practice Guideline for Treating Tobacco Use and Dependence: 2008 Update. A US Public Health Service Report. Am J Prev Med.

[10] Stapleton J, West R, Hajek P (2013). Randomized trial of nicotine replacement therapy (NRT), bupropion and NRT plus bupropion for smoking cessation: effectiveness in clinical practice. Addiction.

[11] Republic of Lebanon - Ministry of Public Health (2015). Mental Health and Substance Use: Prevention, Promotion, and Treatment. Situation Analysis and Strategy for Lebanon 2015-2020.

[12] Karam G, Itani L, Fayyad J, Karam A, Mneimneh Z, Karam E (2016). Prevalence, correlates, and treatment of mental disorders among Lebanese older adults: a national study. Am J Geriatr Psychiatry.

[13] World Health Organization (2011). Developing and improving national toll-free tobacco quit line services: a World Health Organization manual.

[14] Romani M, Jawhar S, Shalak M, Antoun J (2020). Waterpipe smoking cessation: knowledge, barriers, and practices of primary care physicians- a questionnaire-based cross-sectional study. BMC Fam Pract.

